# Signatures in the gut microbiome of German elite athletes: insights from a matched-subgroup analysis

**DOI:** 10.1128/msystems.00489-26

**Published:** 2026-06-30

**Authors:** Claudia Lenz, Waldemar Seel, Theresa Dombrowski, Sebastian Hacker, Marie-Christine Simon, Karen Zentgraf, Christine Dawczynski, Karsten Krüger

**Affiliations:** 1 Department of Exercise Physiology and Sports Therapy, Justus-Liebig-Universität Gießen 9175, Giessen, Germany; 2 Nutrition and Microbiota, Institute of Nutrition and Food Sciences, University of Bonn 9374, Bonn, Germany; 3 Department of Movement and Exercise Science, Goethe-Universität Frankfurt 9173, Frankfurt, Germany; 4 Junior Research Group Nutritional Concepts, Institute of Nutrition, Friedrich Schiller University Jena 9378, Jena, Germany; University of Southampton, Southampton, United Kingdom; SISSA-Trieste, Trieste, Italy

**Keywords:** athletes, healthy adults, matched subgroup, energy metabolism, stress regulation

## Abstract

Elite athletes undergo intense physical training and experience substantial physiological stress, which could affect the composition and function of their gut microbiome. This study compared the gut microbiomes of 148 German junior and senior elite athletes with those of 108 healthy adults to identify taxonomic and functional features associated with elite athletic status. Group comparisons were conducted between healthy adults, senior athletes, and junior athletes, and a matched-subgroup analysis was performed in adults only, controlling for age, sex, body mass index, and dietary pattern. Significant differences in taxonomic composition were observed between athletes and healthy adults. Healthy adults exhibited greater microbial evenness and diversity than junior athletes, whereas senior athletes displayed higher microbial richness. Principal coordinate analysis revealed distinct clustering by athletic status. Linear discriminant analysis effect size identified taxa such as *Escherichia-Shigella* as being enriched in athletes. Predictive metagenomic profiling (PICRUSt2) indicated differences in microbial functional potential between adult athletes and matched controls, including pathways related to amino acid metabolism, glycolysis, fatty acid β-oxidation, and quinone biosynthesis. Together, these findings demonstrate distinct taxonomic and predicted functional microbiome signatures associated with elite athletic status.

**IMPORTANCE:**

Elite athletic training and lifestyle are associated with the gut microbiome. Our research has revealed distinct microbial structures in elite athletes, characterized by reduced evenness in junior athletes and increased richness in senior athletes, compared to healthy adults. Matched-subgroup analyses confirmed these group-specific differences. The gut microbiomes of athletes were enriched in pathways related to amino acid biosynthesis, glycolysis, fatty acid β-oxidation, and quinone synthesis. These microbiome features may be relevant for metabolic efficiency and resilience to oxidative stress. Combining taxonomic and functional prediction data from a uniquely characterized cohort of junior and senior elite athletes provides novel insight into microbiome signatures associated with sustained physical and psychological stress, with potential implications for performance, recovery, and health.

**CLINICAL TRIALS:**

This study is registered with ClinicalTrials.gov as NCT03582020.

## INTRODUCTION

Elite athletes are highly adapted to the performance requirements of their respective sports. While their sport-specific superiority is evident in both preparatory training and competition, the multifactorial conditions underlying long-term performance development and health maintenance continue to be the focus of intensive research. In addition to training and motor skills, factors such as psychosocial influences and cognitive aspects play a crucial role (1). Currently, the role of the gut microbiome in these processes remains to be fully understood. Investigating this is plausible because of the microbiome’s multidirectional association with nutrition, genetics, cognition, metabolism, among others (2).

The gut microbiome has been increasingly studied in regard to physical activity and exercise training (3). There is evidence that the microbial composition may be related to the metabolic, immune, mental, and neurological influence of exercise, thereby potentially impacting various aspects of long-term performance development in athletes (4, 5). In recent studies, it has been suggested that physical activity may promote diversity and the abundance of beneficial bacterial species (6). The gut microbiome of endurance athletes appears to be specifically adapted (7) to accommodate the increased substrate demands associated with their elevated energy metabolism. Short-chain fatty acids (SCFAs), primarily produced through dietary fiber fermentation, play a crucial role in sustaining muscular performance and preserving epithelial integrity (8). Their effects are particularly relevant in mitigating oxidative stress induced by ischemia-reperfusion events in intestinal tissue (6, 9). In addition, adjustments of the microbiome to other physiological processes associated with athletic training could be demonstrated, including the supply of micronutrients and the modulation of immune function (10, 11).

Elite athletes, however, require more than just exceptional endurance or strength and its associated physiological adaptations. They represent a unique population due to their high physical and mental requirements, strict training schedules, and controlled dietary habits (12). Their gut microbiome may reflect specific adaptations to their intense lifestyle and potentially provide insights into how microbiota contribute to performance optimization, recovery, and resilience to physiological and mental stressors (13, 14). In previous studies, different microbial profiles have been found in elite athletes, characterized by changes in microbial diversity and functional pathways associated with energy metabolism and inflammation balance. These findings suggest that the gut microbiome not only has a role in maintaining metabolic performance but may also play a role in protecting athletes from internal and external stressors (5, 13, 15). However, no definitive core set of microorganisms has yet been identified that unambiguously characterizes the gut microbiota of elite athletes from many different disciplines. This challenge arises primarily from the limited access to a large cohort of elite athletes and the complexity of disentangling the differentiated influences of long-term training, dietary habits, and age in high-performance sports (3).

The current study was an investigation of the gut microbiome of German elite athletes from Olympic disciplines such as 3 × 3 basketball, bobsleigh, skeleton, ice hockey, modern pentathlon, gymnastics, table tennis, and volleyball, comparing it to that of healthy adults from the general population. Both cohorts underwent standardized sample collection, gut microbiome sequencing, and metadata analysis to ensure comparability between groups. By examining microbial alpha- and beta-diversity, taxonomic composition, and metabolic pathways, we aimed to identify microbial features associated with high physical training demands and high performance of athletes. To further disentangle the effects of athletic status from potential confounding variables, we conducted a matched-subgroup analysis controlling for age, body mass index, and dietary patterns to better characterize microbiome signatures associated with elite athletic requirements. Based on relevant findings from previous studies in athletes, it was hypothesized that elite athletes exhibit a distinct microbiome profile, characterized by differences in microbial diversity and composition associated with sport-specific physiological requirements. These findings contribute to a deeper understanding of the gut microbiome as a key physiological system potentially relevant for long-term performance development in athletes.

## MATERIALS AND METHODS

### Study design and data collection

**Fig 1:**
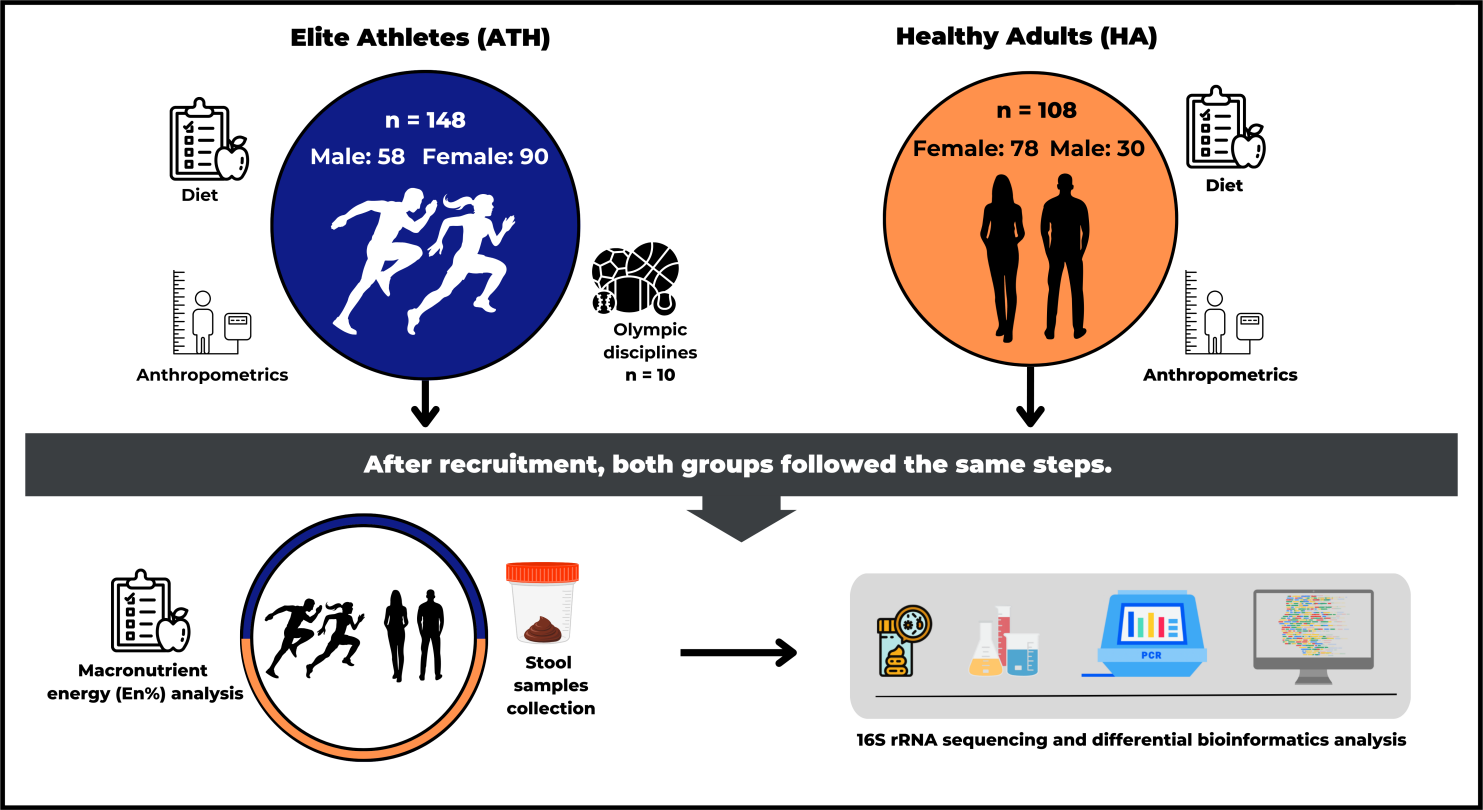
Overview of the study cohort, including elite athletes (ATH) from the in:prove project and healthy adults (HA) from the NuEva project. The figure also summarizes the available metadata, data collection, and workflow process.

The study cohorts were recruited from two projects, the NuEva and in:prove study (Fig. 1). The NuEva cohort comprised healthy adults (HA), whereas the in:prove project specifically targeted German elite athletes (ATH), with an emphasis on individualized aspects of performance.

The demographic and background information of the study population is detailed in Table 1. For this work, a group of 148 German elite athletes (90 women) participating in in:prove were tested, ranging in age from 13 to 39 years, with an average age of 19.3 years. The participants represented ten different Olympic disciplines: 3 × 3 basketball, gymnastics, rhythmic gymnastics, trampoline gymnastics, bobsleigh, skeleton, ice hockey, modern pentathlon, table tennis, and volleyball. Elite athletes were defined as active members of German national squads (junior, perspective, or Olympic squads) across Olympic-level sports disciplines, encompassing endurance-based (e.g., modern pentathlon), strength/power (e.g., bobsleigh), and skill-based (e.g., table tennis) profiles. While acknowledging the heterogeneity of exertion demands across these sports, this broad definition reflects the diversity of elite-level performance in high-performance sport. No participants were excluded based on antibiotic use, probiotics, prebiotics, supplements, or chronic medication. Supplement use was systematically recorded and considered during subgroup selection to ensure comparability but was not included in statistical models due to incomplete data coverage.

**TABLE 1:** Descriptive characteristics of athletes (ATH and ATHu18, classified as over and under 18 years) and healthy adults (HA), as well as energy and macronutrient consumption^*a*^

Parameter	HA (*n* = 108)	ATH (*n* = 82)	ATHu18 (*n* = 66)	*P*-value	Post-hoc
Age (years)	35.0 ± 12.01	23.2 ± 4.3	15.5 ± 1.24	0.001	HA vs. ATH *P* < 0.001; HA vs. ATH-u18 *P* < 0.001; ATH vs. ATH-u18 *P* < 0.001
BMI (kg/m^2^)	24.16 ± 3.85	23.70 ± 3.0	21.24 ± 2.21	0.001	HA vs. ATH-u18 *P* < 0.001; ATH vs. ATH-u18 *P* < 0.001
Weight (kg)	70.81 ± 13.37	76.42 ± 14.56	66.36 ± 12.31	0.001	ATH vs. HA *P* 0.014; ATH vs. ATH-u18 *P* < 0.001
Total energy consumption (kcal)	2,391.88 ± 797.37	2,474.51 ± 832.24	2,541.08 ± 848.62	0.496	
Carbohydrates (E%)	45.07 ± 8.71	45.97 ± 6.65	48.74 ± 7.11	0.009	HA vs. ATH-u18 *P* < 0.009
Lipids (E%)	34.03 ± 6.11	31.86 ± 6.66	32.34 ± 7.08	0.058	
Proteins (E%)	15.83 ± 3.35	18.07 ± 4.29	16.95 ± 3.22	0.001	HA vs. ATH *P* < 0.001

^*a*^ Data are presented as the means ± standard deviation. HA, healthy adults; ATH, elite athletes; ATHu18, junior athletes.

The NuEva study was conducted in Central Germany (recruitment area: Central Eastern Germany, Jena-Halle-Leipzig). Vegans, vegetarians, flexitarians, and omnivores took part in the long-term study in a parallel design with an allocation ratio of 1:1:1:1. The study began in 2018; the follow-up phase ended in December 2020 (16). A total of 108 flexitarians/omnivores (78 women) aged 20–69 years (35.2 ± 12.1) from the NuEva collective were included in the present analysis. The data from nutrition logs and stool samples for this study were collected at the beginning of the NuEva study. To minimize potential confounding, a matched subgroup of elite athletes (ATHsub, *n* = 20; 10 female, 10 male) and healthy adults (HAsub, *n* = 21; 12 female, 9 male) was selected from the full cohorts. This subgroup represents a frequency-matched cohort based on sex (balanced at the group level), age (± 3 years), body mass index (± 2 kg/m²), and dietary pattern. Exact matching was applied at the variable level; however, strict 1:1 pairing was not enforced in order to retain sufficient sample size under the given constraints. Selection of the matched subgroup was restricted to participants with complete microbiome, anthropometric, and dietary data and was conducted independently of microbiome composition or outcome variables. Matching quality was assessed descriptively; no meaningful imbalances in matching variables were observed between groups. In the matched subgroup, ATHsub samples were distributed across six sequencing runs, whereas HAsub samples originated from a separate batch; thus, no sequencing run contained samples from both cohorts. Residual batch/run effects cannot be excluded. Details on sequencing structure are provided in “16S rRNA library preparation and sequencing,” below.

### Dietary assessment

All participants (NuEva and in:prove) documented their individual dietary intake for 3–5 days parallel to the collection of stool samples. For NuEva participants, full self-reports on intake of foods and beverages (type and quantity) were completed over five days in the run-in period. The dietary record was based on the template “Freiburger Ernährungsprotokoll,” which was provided by PRODI version 6.4 (Nutri-Science, Stuttgart, Germany) and includes common foods and usual portion sizes. To obtain individual information, missing food items could be added. Foods that were basically not contained in PRODI were created and the nutritional information was taken from the packaging (including fortification with vitamin B12 and calcium). The daily energy and nutrient intake were calculated by the software package PRODI. The nutrient intake from supplements was not considered in the calculation of nutrient intake by the dietary protocols (16).

In a comparable manner, in:prove athletes were instructed and briefed on the accurate documenting of all foods, beverages, and supplement consumption, including the type and quantities of each, in a 3-day dietary protocol (17, 18). The protocols were then analyzed using the DGExpert software (version 2.0.37). The Foodlog software PRODI and DGExpert are both based on the German Food Code and Nutrient Database (BLS). Energy in percent (En%) for macronutrients was calculated for all participants. This approach reduces methodological limitations regarding the comparability of dietary data between cohorts. For the matched-subgroup analysis, dietary matching was limited to dietary pattern (omnivorous diet), while quantitative dietary variables (total energy intake, macronutrient distribution, and fiber intake) were not used as matching criteria but were derived as averaged daily values for descriptive and adjustment purposes.

### Gut microbiome analysis

#### DNA extraction

Genomic DNA was extracted from 120 mg fecal samples using the ZR BashingBead lysis tubes (0.1 and 0.5 mm, Zymo Research, Freiburg, Germany) in conjunction with the chemagic DNA Stool 200 Kit H96 (Perkin Elmer, Rodgau, Germany), following the manufacturer’s protocol (19–21). A mechanical cell lysis step was performed after adding the lysis buffer using the Precellys 24 Tissue Homogenizer (Bertin Instruments, Frankfurt am Main, Germany). Following extraction, the obtained DNA was stored at −20°C until further use.

#### 16S rRNA library preparation and sequencing

Amplicon sequencing of the fecal microbiome was conducted by Life & Brain (Bonn, Germany). In brief, the V3V4 region of the 16S rRNA gene was amplified in the first PCR step using the primer Bakt_341F (5′-CCTACGGGNGGCWGCAG-3′) and Bakt_805R (5′-GACTACHVGGGTATCTAATCC-3′) and subsequently pooled equimolarly at 4 nM as outlined in previous research (22). The final pooled sample was quantified using the Qubit dsDNA HS Assay Kit from Thermo Fisher Scientific (Waltham, MA, USA), and fragment size was verified using a D1000 ScreenTape. Sequencing was performed on an Illumina MiSeq system with the MiSeq Reagent Kit v3, utilizing 2 × 300 cycles. Clustering was carried out at 6 pM with a 15% Phi-X spike-in. Demultiplexing was performed on the MiSeq system. Sequencing was performed across multiple runs at different time points. In the matched subgroup, ATHsub samples were distributed across six sequencing runs, whereas HAsub samples originated from a separate batch. Accordingly, no sequencing run contained samples from both cohorts. At the same time, processing and sequencing were not performed in completely isolated phases but showed partial temporal interspersion. No negative controls (e.g., extraction blanks or mock communities) were included in the sequencing workflow. Because sequencing run/batch was structurally confounded with cohort/project-of-origin in the matched subgroup, a formal sensitivity analysis including sequencing run as a covariate, or restriction to shared runs, was not considered statistically meaningful. Residual batch/run effects therefore cannot be excluded and were considered in the interpretation of subgroup differences.

### Data analysis

Data sets for participants recruited from both projects were merged; in addition, unit conversions were performed when necessary. Implausible values were set to “missing” after health, diet, and blood variables were checked for outliers. Group differences were assessed using an independent *t*-test (for two groups) or one-way ANOVA (for three groups), with post-hoc Tukey tests if ANOVA was significant. Normality was checked via the Shapiro-Wilk test, and Levene’s test was used to assess for the homogeneity of variances. Non-parametric Kruskal-Wallis tests were used when assumptions were violated. Pairwise comparisons following significant Kruskal-Wallis tests were performed using Dunn’s test with Bonferroni correction. All reported *P*-values for pairwise comparisons are adjusted *P*-values. FDR correction was applied separately for each alpha-diversity metric across all pairwise group comparisons.

The 16S sequencing data were analyzed using QIIME 2 version 2024.2 (23). In short, sequence quality control and denoising were performed using DADA2 (24). After quality filtering, joining, and chimera removal, between 52% and 79% of the raw reads remained. The quality-filtered sequences were then classified using the SILVA SSU 138 database to identify amplicon sequencing variants with >99% sequence similarity.

To obtain core metrics, a rarefied table was used to calculate the following alpha-diversity metrics: observed features, Shannon index, Pielou’s evenness, and Faith-PD. Beta-diversity metrics were also calculated, including Bray-Curtis, Jaccard, and (un)weighted UniFrac. Samples were rarefied to a depth of 18,153 reads for both analyses to ensure comparable sequencing depth. This threshold was chosen based on rarefaction curves to retain the majority of samples while preserving sufficient coverage of community diversity. Following this step, *n* = 4 (two from each cohort) samples with fewer reads were excluded from diversity analyses. PERMANOVA analyses were performed using the adonis function with 999 permutations and the model defined as: distance matrix ~group + age + sex + fiber intake. The primary PERMANOVA analysis compared ATH with HA. Analyses including junior athletes ATHu18 were conducted separately and are indicated accordingly in Results.

**Fig 2:**
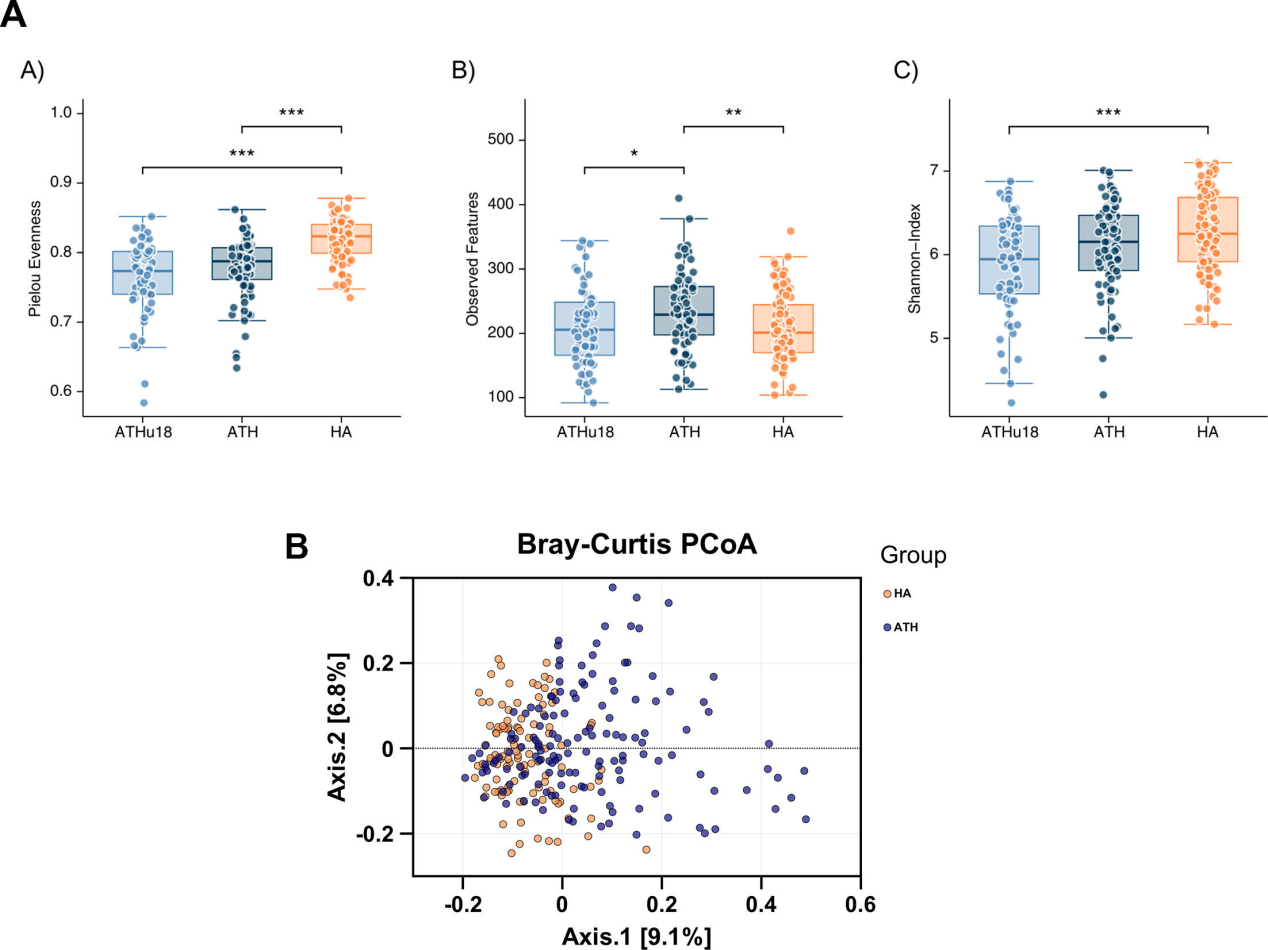
Alpha-diversity and beta-diversity of the overall sample. (**A**) Alpha-diversity analysis for healthy adults (HA) and elite athletes (ATH and ATHu18, classified as over and under 18 years), including (A) evenness, (B) observed species (OBS), and (C) Shannon index. Statistical significance levels: *P < 0.05, **P < 0.01, ***P < 0.001. Values indicate the level of significance for the comparison between groups. (**B**) Bray-Curtis PCoA, with axes representing the percentage of variation explained by each coordinate dimension.

**Fig 3:**
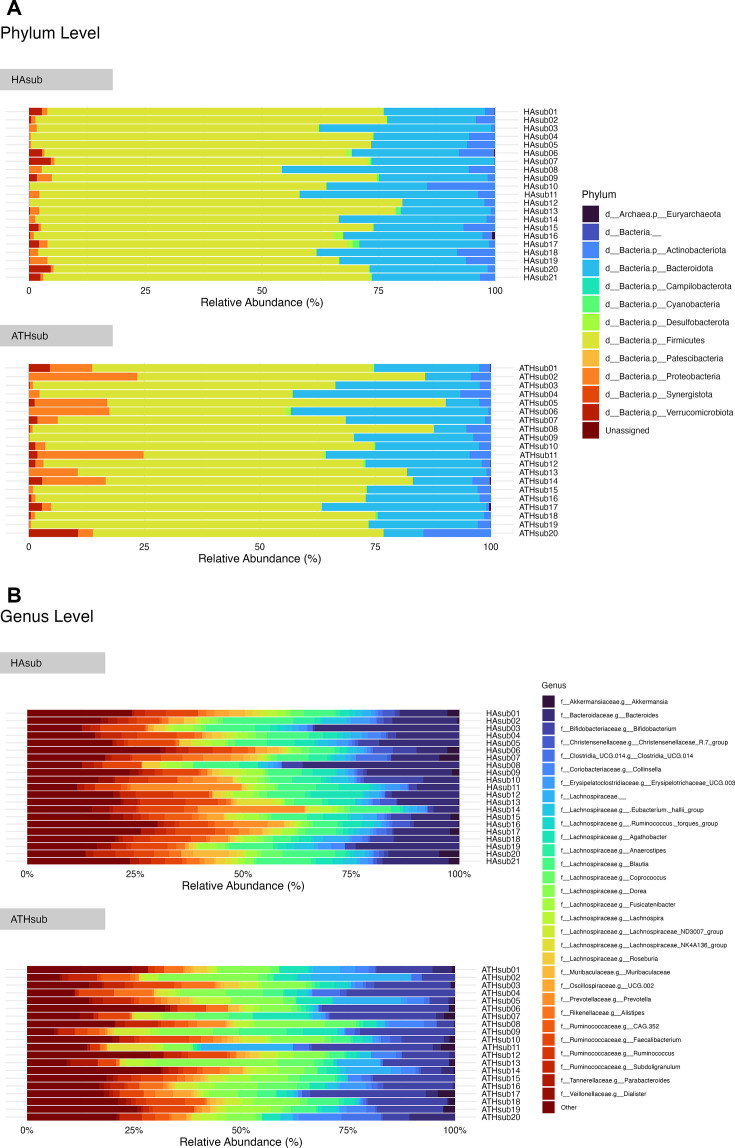
Overview of the relative abundance in the matched subsample at the phylum (**A**) and genus (**B**) levels. Athletes are classified as ATHsub, and healthy adults as HAsub. The genus-level analysis displays the 30 most abundant genera.

Matched-subgroup analyses were performed using participants with comparable age, body mass index, and diet, thereby minimizing non-biological sources of variation. Given the frequency-matched design without individual pairing, all subgroup analyses were conducted using unpaired statistical tests. The subset samples were treated in the same way, with the following analyses performed in addition. R “vegan” package (version 2.6-10) was used to calculate adjusted *P*-values, controlling for age, sex, and fiber intake for PERMANOVA analysis of beta-diversity metrics (adonis2). The model was defined as diversity matrix ~group + age + sex + fiber intake (LMMadj.) Standard table filter settings were used in Microbiome Analyst (25) to perform linear discriminant analysis effect size (LEfSe) analysis. LEfSe analysis was used as an exploratory approach and should be interpreted as hypothesis-generating rather than confirmatory. The Phylogenetic Investigation of Communities by Reconstruction of Unobserved States 2 (PICRUSt2, version 2.5.1) was used to predict the functional potential of the fecal microbiota (26). Predicted pathways in each sample were annotated using the MetaCyc database (27). Both statistical and graphical analyses were performed using STAMP v2.1.3 (28). Figure 1 was created using Canva (version 1.103.0, Canva Pty Ltd, Surry Hills, New South Wales, Australia). Figure 2A and 3 were created using R (version 4.4.2; R Core Team; R Foundation for Statistical Computing, Vienna, Austria) and RStudio (version 2024.4.2.764; Posit Team; Posit Software, PBC, Boston, MA, USA), using the packages ggplot2 and patchwork.

## RESULTS

A total of 256 participants were included in the analysis. The sample consisted of 108 healthy adults (HA), 82 athletes (ATH) over 18 years, and 66 junior athletes (ATHu18). Total energy consumption (kcal) did not differ between groups, averaging 2391.88 (± 797.37) in HA and 2474.51 (± 832.24) in ATH. Similarly, fiber intake (g/day) showed no significant differences among the three cohorts, ranging from 27.90 (± 10.51) in HA to 30.83 (± 12.68) in ATH. All macronutrient, fiber, and energy data are presented in Table 1.

### Alpha-diversity differences between adult athletes (ATH), junior athletes (ATHu18), and healthy adults (HA)

All reported *P*-values for pairwise comparisons are adjusted *P*-values following FDR correction. The analysis of alpha-diversity metrics revealed significant differences between the three cohorts (Kruskal-Wallis test). For evenness, HA demonstrated significantly higher evenness compared to both ATH and junior athletes ATHu18 (*P* < 0.001), indicating a more balanced microbial community. No significant difference was observed between ATH and ATHu18. In terms of observed features (OBS), ATH displayed a significantly richer microbial community compared to HA (*P* = 0.007) and ATHu18 (*P* = 0.033). The Shannon index, which reflects both richness and evenness, was significantly higher in HA compared to ATHu18 (*P* < 0.001). No significant differences were found between HA and ATH, with a tendency to a higher Shannon index in HA (Fig. 2).

Principal coordinates analysis (PCoA) based on the Bray-Curtis dissimilarity metric revealed distinct clustering of ATH and HA groups, indicating significant differences in microbial community composition (Fig. 2B), which was statistically supported by PERMANOVA results (Table 2). The first two principal coordinates accounted for the majority of variation, with clear separation between the groups. Subgroup analysis further demonstrated a consistent clustering pattern in ATHsub and HAsub.

**TABLE 2:** PERMANOVA results for weighted UniFrac and Bray-Curtis dissimilarity metrics comparing adult athletes (ATH) and healthy adults (HA)^*a*^

Metric	Df	SumsOfSqs	MeanSqs	F.model	R^2^	*P*-value
Weighted UniFrac						
Age	1	0.337	0.337	12.945	0.047	0.001
Sex	1	0.031	0.031	1.182	0.004	0.270
Fiber intake	1	0.064	0.064	2.450	0.009	.025
Group (ATH/HA)	1	0.250	0.250	9.593	0.035	0.001
Residuals	247	6.438	0.026	NA^*b*^	0.904	NA
Total	251	7.121	NA	NA	1.000	NA
Bray-Curtis						
Age	1	1.174	1.174	4.584	0.018	0.001
Sex	1	0.332	0.332	1.294	0.005	0.054
Fiber intake	1	0.687	0.687	2.682	0.010	0.001
Group (ATH/HA)	1	0.900	0.900	3.515	0.014	0.001
Residuals	247	63.279	0.256	NA	0.953	NA
Total	251	66.372	NA	NA	1.000	NA

^*a*^ Analyses were performed using the adonis function with 999 permutations and the model: distance matrix ~ group + age + sex + fiber intake. The table reports degrees of freedom (Df), sum of squares (SumsOfSqs), mean squares (MeanSqs), F-values (F.Model), proportion of variance explained (R²), and *P*-values [Pr(>F)].

^*b*^ NA, not applicable.

### Beta-diversity comparisons between athletes (ATH/ATHu18) and healthy adults

Microbial beta-diversity significantly differed between ATH and HA, as assessed by Bray-Curtis dissimilarity and weighted UniFrac distances (Table 2). Analyses including ATHu18 are reported separately where applicable. PERMANOVA analysis revealed that group membership (ATH vs. HA) was a significant factor explaining microbial composition variance in both metrics (Bray-Curtis: R^2^ = 0.014, *P* = 0.001; weighted UniFrac: R^2^ = 0.035, *P* = 0.001). Among potential confounding factors, age was found to have the strongest effect on microbial composition (Bray-Curtis: R^2^ = 0.018, *P* = 0.001; weighted UniFrac: R^2^ = 0.047, *P* = 0.001), while fiber intake showed a weaker but still significant association (Bray-Curtis: R^2^ = 0.010, *P* = 0.001; weighted UniFrac: R^2^ = 0.009, *P* = 0.025). In contrast, sex had no significant effect on microbial beta-diversity in either analysis (Bray-Curtis: R^2^ = 0.005, *P* = 0.054; weighted UniFrac: R^2^ = 0.004, *P* = 0.270).

### Matched-subgroup comparison between adult athletes (ATHsub) and healthy adults (HAsub)

This subgroup analysis was restricted to adult participants to allow for robust matching by age, body mass index, and dietary pattern. A comparable matched non-athletic adolescent control group was not available; therefore, no junior athletes subgroup was conducted. Descriptive characteristics of the HAsub and ATHsub subgroups are presented in Table 3. No significant differences were observed between groups in terms of age or BMI. Both groups followed an omnivorous diet, with similar total energy intake and macronutrient distribution. Figure 3 illustrates the relative abundances of microbial taxa at both the phylum and genus levels. At the phylum level, a higher prevalence of *Proteobacteria* and *Actinobacteriota* was found in ATHsub. The relative abundances of other phyla remained relatively stable across both groups, with individual variations (Fig. 3A). At the genus level, ATHsub exhibited a higher relative abundance of *Lactobacillus* and *Bifidobacterium* genera, while HAsub showed a greater abundance of *Bacteroides* and *Prevotella* (Fig. 3B).

**TABLE 3:** Descriptive characteristics of the HAsub (healthy adults) and ATHsub (elite athletes) subgroups

Parameter	HAsub (*n* = 21)	ATHsub (*n* = 20)	*P*-value
Age (years)	30.8 ± 3.8	29.0 ± 2.6	0.078
BMI (kg/m^2^)	24.32 ± 3.6	25.49 ± 3.58	0.303
Weight (kg)	75.08 ± 13.68	83.45 ± 17.09	0.090
Diet	Omnivore	Omnivore	
Total energy consumption (kcal)	2,691.41 ± 845.14	2,543.77 ± 799.21	0.651
Carbohydrates (En%)	42.69 ± 8.57	44.40 ± 8.29	0.519
Lipids (En%)	35.63 ± 5.79	32.82 ± 7.13	0.173
Proteins (En%)	17.82 ± 4.9	17.67 ± 3.38	0.382
Fiber (g/day)	29.51 ± 11.42	31.36 ± 12.39	0.625

### Microbial differences between ATHsub and HAsub

**Fig 4:**
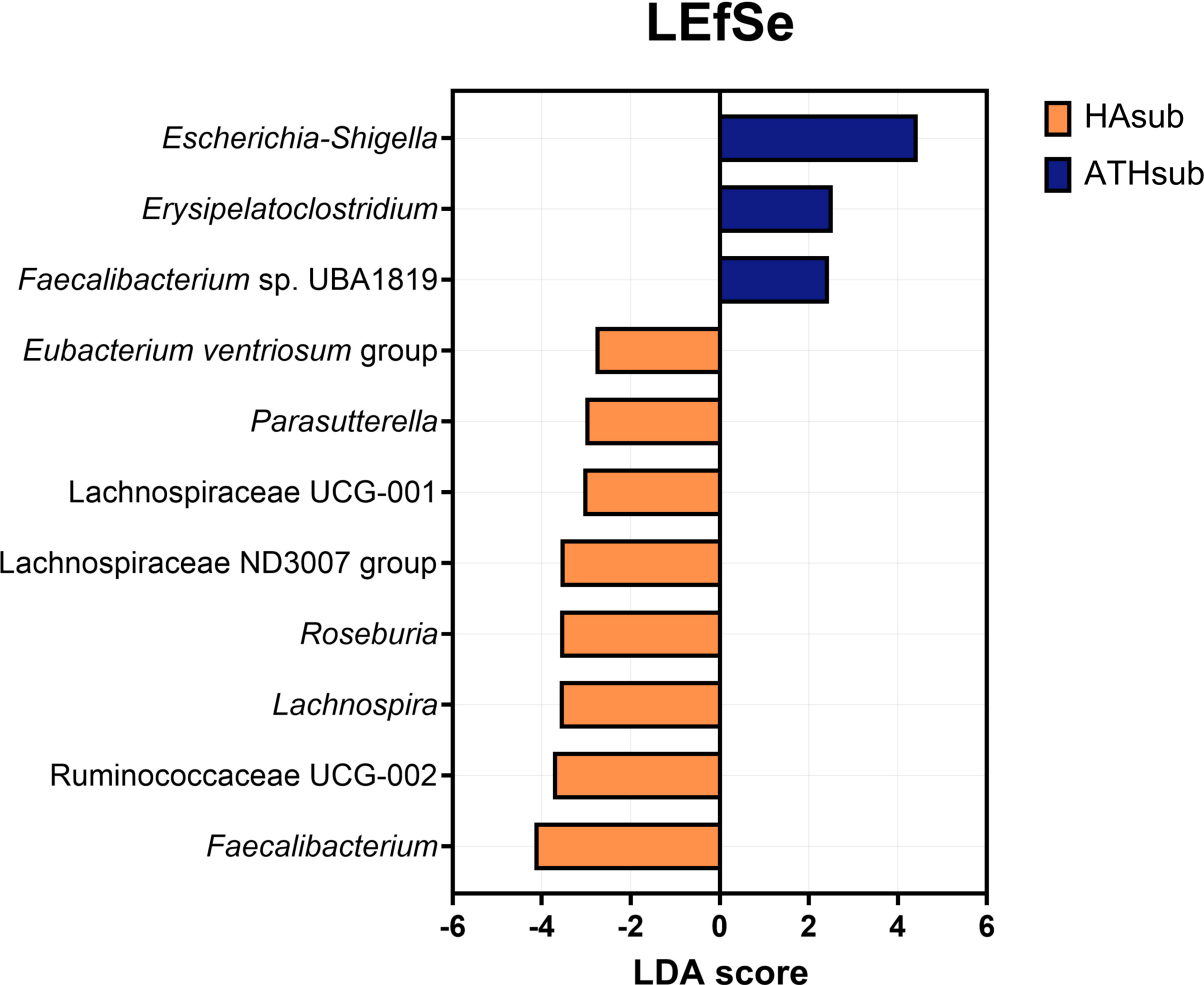
The results from LEfSe analysis showing the top 11 taxa that significantly differ between elite athletes (ATHsub) and healthy adults (HAsub). All the enriched taxa have a minimum LDA score of 2, indicating that these taxa are at least twice as abundant in the respective group.

The LEfSe analysis (<0.05) was performed using an alpha level of 0.05 and a linear discriminant analysis (LDA) score threshold of >2.0 to identify taxa with significant differences in abundance between the groups. In ATHsub, enriched taxa included *Escherichia-Shigella*, *Erysipelatoclostridium*, and the genus *Faecalibacterium* sp. UBA1819. These taxa exhibited the highest LDA scores, suggesting a strong association with the ATHsub. In contrast, the HAsub were significantly enriched in several genera, including *Faecalibacterium*, *Roseburia*, *Lachnospira*, and *Parasutterella*. Notable members of the Lachnospiraceae family, such as *Lachnospiraceae_UCG_001* and *Lachnospiraceae_ND3007*, also contributed significantly to the microbial profile of the HAsub (Fig. 4).

### Predictive functional differences between the matched subgroups

**Fig 5:**
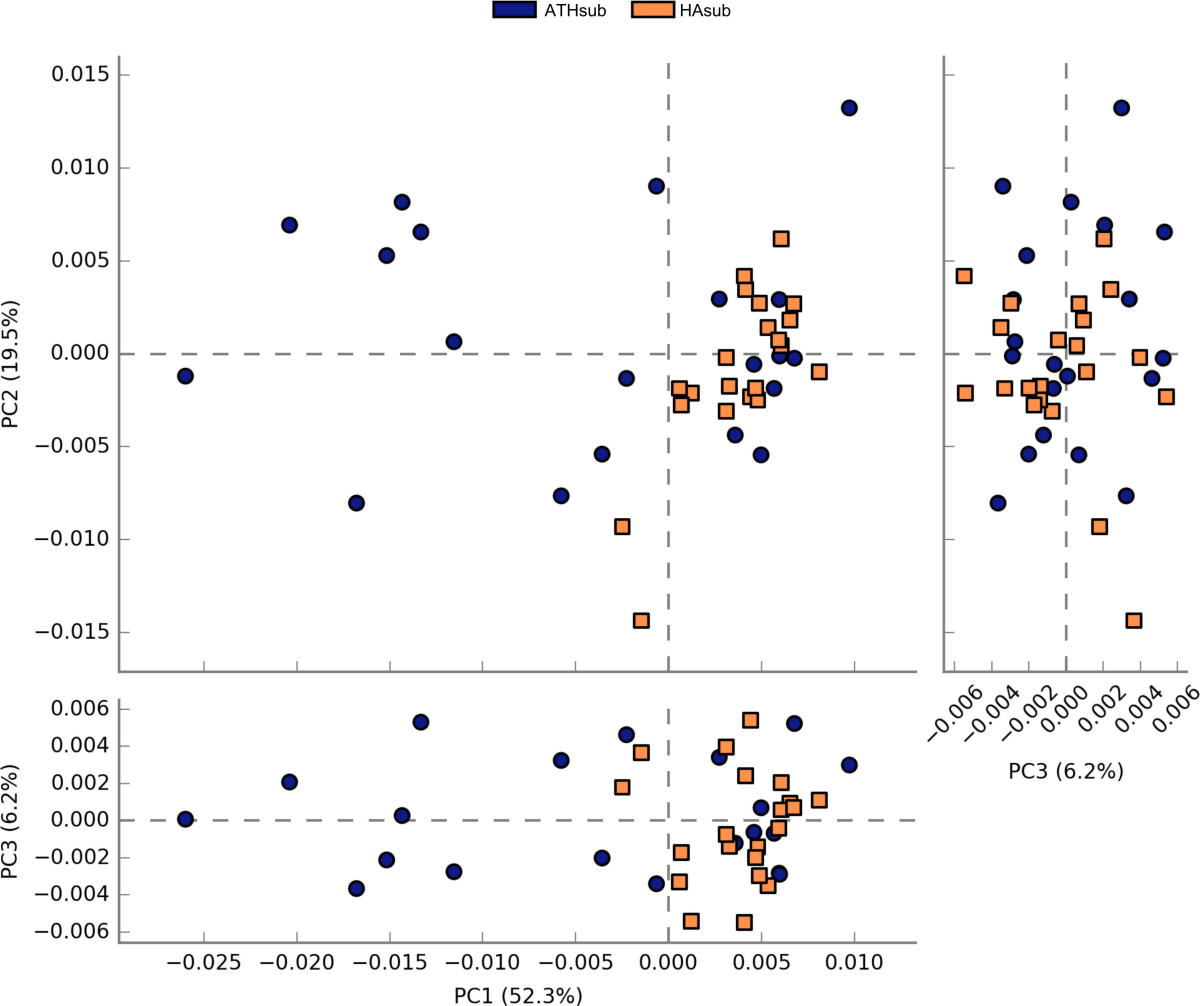
PCoA based on beta-diversity metrics for HAsub and ATHsub. The PCoA plot displays the distribution of samples along the first three principal coordinates (PC1, PC2, and PC3). Dashed lines represent the origin of each axis. The plot illustrates the separation and clustering patterns between groups (ATHsub and HAsub) based on microbial community composition.

To elucidate predicted functional differences in microbial metabolic capacity between ATHsub and HAsub, a predictive metagenomic profiling approach based on 16S rRNA gene data was applied using PICRUSt2. A PCoA based on Bray-Curtis dissimilarity was performed (Fig. 5). The first principal component (PC1) explained 52.3% of the variance, followed by PC2 (19.5%) and PC3 (6.2%). The ordination plot revealed a clear separation between the two groups, particularly along PC1, suggesting distinct microbial functional capacity profiles. The data for ATHsub individuals tended to cluster separately from HAsub individuals, with ATHsub samples showing greater dispersion.

**Fig 6:**
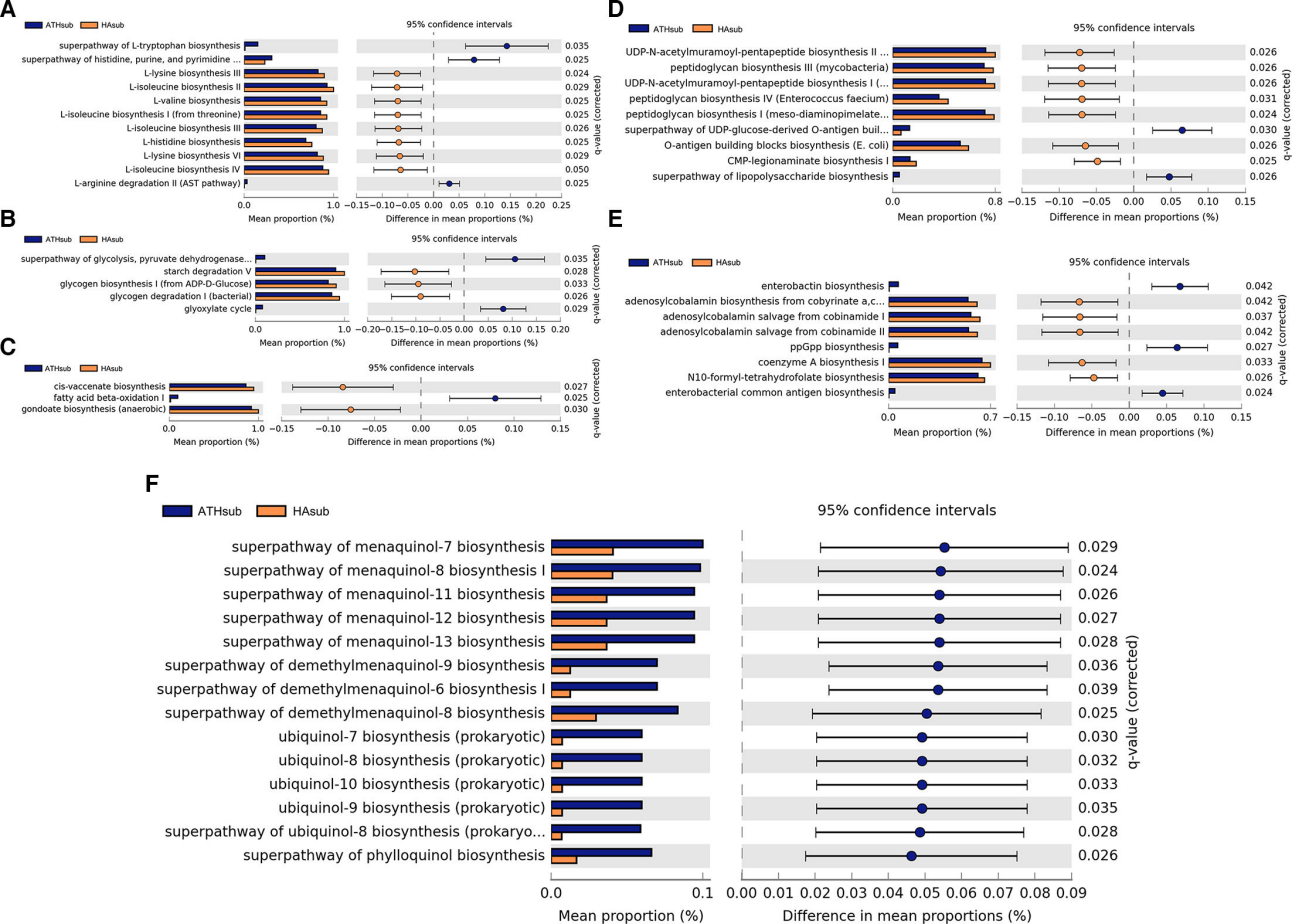
Predicted functional microbiome differences between elite athletes (ATHsub) and healthy adults (HAsub) based on PICRUSt2 analysis**.** Mean relative abundances (%) of significantly different MetaCyc pathways are shown with 95% confidence intervals and FDR-corrected q-values. (**A**) Amino acid metabolism pathways; (**B**) carbohydrate metabolism pathways; (**C**) lipid metabolism pathways; (**D**) bacterial cell wall and structural biosynthesis pathways; (**E**) coenzyme and vitamin biosynthesis pathways; and (**F**) quinone biosynthesis and electron transport pathways.

PICRUSt2 analysis revealed distinct metabolic capacity signatures between the two groups, with significant differences in pathways related to amino acid metabolism, carbohydrate metabolism, lipid metabolism, and biosynthesis of structural and coenzyme components (Fig. 6).

Pathways related to amino acid biosynthesis (Fig. 6A), particularly L-lysine, L-isoleucine, L-valine, and L-histidine, were significantly enriched in HAsub. In contrast, pathways involved in L-tryptophan and the superpathway of histidine, purine, and pyrimidine biosynthesis were elevated in ATHsub. The L-arginine degradation pathway, which plays a role in nitrogen metabolism and nitric oxide production, was also more prevalent in ATHsub.

The superpathway of glycolysis, pyruvate dehydrogenase, and the glyoxylate cycle were enriched in ATHsub compared to HAsub. HAsub was significantly more enriched in the starch degradation pathway V and glycogen biosynthesis I than ATHsub (Fig. 6B). Differences in lipid metabolism were evident, with ATHsub showing higher proportions of pathways related to fatty acid β-oxidation (Fig. 6C). The results presented in Fig. 6D indicate distinct differences in bacterial cell wall composition between the groups. The lipopolysaccharide biosynthesis superpathways were significantly more abundant in ATHsub, while several peptidoglycan biosynthetic pathways, including UDP-N-acetylmuramoyl-pentapeptide biosynthesis and mycobacterial peptidoglycan synthesis, were significantly enriched in HAsub. Regarding microbial functional profiling, HAsub exhibited a significantly higher mean proportion of adenosylcobalamin (vitamin B12) biosynthesis and salvage pathways, including biosynthesis from cobyrinate and salvage from cobinamide I and II (Fig. 6E). The pathway for ppGpp biosynthesis, a key regulator of bacterial stress responses and metabolic adaptation, was significantly more abundant in ATHsub.

In terms of the functional capacity of the quinone biosynthesis and electron transport pathways, all pathways were more abundant in ATHsub (Fig. 6F). A significantly higher abundance of menaquinol (vitamin K2) and ubiquinol biosynthesis pathways was exhibited in ATHsub compared to HAsub. Multiple menaquinol biosynthesis pathways, such as menaquinol-7 to menaquinol-13, were represented more often in ATHsub. Similarly, demethylmenaquinol and ubiquinol biosynthesis pathways, such as ubiquinol-7 to ubiquinol-10, were more prevalent. Additionally, phylloquinol (vitamin K1) biosynthesis was elevated in ATHsub (Fig. 6F).

## DISCUSSION

In this study, we demonstrated significant structural and predicted functional differences in the gut microbiome of elite athletes compared with healthy adults. These differences are consistent with training- and lifestyle-associated effects on the gut microbiome. However, given the cross-sectional design of the study, causal relationships cannot be established, and the observed microbial signatures may also reflect pre-existing microbiome characteristics that predispose individuals to elite athletic performance. When comparing athletes (ATH) and HA, HA showed greater evenness and ATH a richer microbiome (OBS), but no significant differences in Shannon index. Junior athletes and HA were significantly different in terms of evenness and Shannon index. A lack of significant differences between ATH and HA suggests that age-related microbiome stabilization may have a more pronounced effect than training alone. Beta-diversity differed between groups, even after adjusting for age and fiber intake, which were significantly associated with microbial composition. However, these variables did not fully account for the observed differences between ATH and HA. Subgroup analysis revealed that ATHsub exhibited higher levels of *Firmicutes* and *Proteobacteria,* whereas HAsub showed a greater prevalence of *Bacteroidota*, possibly reflecting differences in metabolic demands. LEfSe analysis indicated significant differences in microbial abundance, with ATHsub showing enriched levels of *Escherichia-Shigella, Erysipelatoclostridium,* and *Faecalibacterium sp.* UBA1819. These findings might represent adaptations driven by the athletes’ high training volume. Predictive functional profiling was used to identify enhanced L-arginine degradation in ATHsub, suggesting greater metabolic efficiency. Interestingly, HAsub have a higher functional capacity for essential amino acids such as L-lysine, L-isoleucine, L-valine, and L-histidine. This may indicate that ATHsub have higher requirements, resulting in reduced availability of these amino acids as an educt. Differences in metabolic capacity included increased functional glycolysis and β-oxidation pathways in ATHsub. Additionally, pathways involved in cell wall and coenzyme biosynthesis were enriched, accompanied by enhanced bacterial stress responses and increased menaquinol/ubiquinol biosynthesis, suggesting functional microbial adaptations that may influence vitamin K synthesis in ATHsub. The observed microbiome characteristics may reflect both training-related influences and pre-existing microbial traits that could predispose individuals to elite athletic performance.

### Microbial diversity at alpha and beta levels

The observed differences in gut microbiome alpha-diversity metrics between ATH, ATHu18, and HA may provide insights into the relationship between regular intensive exercise training, age, and microbial diversity. Although ATH showed greater richness compared to HA, this suggests that long-term intensive exercise, increased energy demands, and specialized diets may play a key role in shaping microbiota composition (12). The long-term effect can be supported by the difference between ATH and ATHu18, as well as the absence of differences between HA and ATHu18. However, the influence of age shaping the microbiome must be considered (29). Richness, often associated with a diverse and resilient gut environment, may provide competitive advantages for athletes by optimizing metabolic flexibility, immune regulation, and recovery processes (30). In contrast, HA demonstrate greater evenness compared to ATH and ATHu18, suggesting that microbial species are more evenly distributed in HA. In ATH and ATHu18, certain taxa dominate the microbiome, and these taxa could be specific to high levels of physical activity and metabolism (31). The lower evenness and Shannon index suggest athletes’ microbiome is enriched in specific taxa beneficial for high-performance metabolism. These findings align with the results of previous studies, suggesting that a diverse gut microbiome may support the physiological demands of elite sports via enhanced energy metabolism and immune-regulating properties (32). Other authors have also shown that elite athletes tend to have greater microbial diversity than controls (15, 33). In line with these findings, studies in elite rugby players and endurance athletes have further reported exercise-associated shifts in gut microbiome composition, including increased microbial diversity and enrichment of taxa such as *Akkermansia* and members of the *Firmicutes* phylum (15). Differences between these studies and the present findings likely reflect variation in sport modality, training volume, dietary practices, and analytical design, including the use of matched-subgroup approaches. However, it is crucial to acknowledge that the HA population of the study consisted of metabolically healthy adults, which may serve as a potential explanation for the observed absence of significant differences (34). Hintikka et al. analyzed a matched cohort of elite cross-country skiers and healthy controls, finding no significant differences in alpha-diversity, as indicated by comparable Shannon index and Chao1 values (35). These findings are consistent with previous studies in which it was shown that overall microbial diversity in adults remains largely stable regardless of training intensity or dietary variations (15, 36). Together, these findings highlight that athlete-control differences in gut microbiome composition are not uniform across cohorts and may depend on age, training history, sport modality, and analytical design, including matching strategies.

PERMANOVA analysis based on Bray-Curtis dissimilarity and weighted UniFrac highlighted significant differences in beta-diversity between study groups, with age and group emerging as the key factors contributing to variations in microbial community composition. Age emerged as the strongest predictor of beta-diversity, highlighting its significant influence on gut microbiota composition. This finding aligns with the results of prior research, suggesting that age-related changes in the gut microbiome reflect cumulative lifestyle factors, dietary patterns, and physiological adaptations over time (29, 37). These changes likely promote microbial stabilization and diversification in adulthood, as reflected in higher alpha-diversity in ATH and HA compared to junior athletes. Group membership significantly shaped microbial composition, highlighting distinct signatures associated with elite athletic status versus healthy individuals. The overlap in microbial communities between adult athletes and healthy adults may result from their shared influences of training and age. In contrast, junior athletes might exhibit distinct profiles due to their younger age and variations in training history, some having trained at an elite level for years, while others began structured training only in adolescence (38). The minor, non-significant effect of sex aligns with previous findings, suggesting that microbiome differences between sexes are relatively small and more influenced by factors such as lifestyle or diet (39, 40). A high proportion of residual variance highlights the complexity of factors influencing beta-diversity beyond those explicitly tested in the model.

### Microbiota composition in matched subgroups

The results of in-depth research using age, sex, and diet-matched subgroup analysis in this study suggest that there are adaptations in the gut microbiome of German elite athletes. At the phylum level, ATHsub exhibited an enrichment of *Firmicutes* and *Proteobacteria*, whereas *Bacteroidota* predominated in HAsub. This finding aligns with the results of Han et al., who reported that variations in the relative abundance of *Firmicutes* and *Bacteroidota* are influenced by factors such as diet and training status (41). A potential adaptive response to elevated physical demands may be indicated by an increased presence of *Proteobacteria* in athletes. This phylum encompasses taxa involved in energy metabolism and oxidative stress regulation (42). However, greater *Proteobacteria* levels could also reflect a microbiota under stress, warranting further functional analysis to determine whether this shift represents a beneficial adaptation or a potential risk factor for dysbiosis (43). Increased relative abundance of *Actinobacteriota* in ATHsub aligns with their known role in gut health and immune function, potentially supporting the heightened metabolic demands and anti-inflammatory requirements of elite athletes (44). The overall stability of major phyla between groups, despite individual variability, suggests that while athletic training induces specific microbial shifts, core microbial characteristics remain resilient to lifestyle differences. The increased prevalence of *Lactobacillus* and *Bifidobacterium* in ATHsub suggests a microbiota adapted to physical stress and performance. *Bifidobacterium* is known for strengthening the intestinal barrier by supporting tight junctions between epithelial cells, thus reducing the risk of “leaky gut” (45), which is challenged during intense training. It also contributes to increased fermentation of dietary fiber and SCFAs production (3, 46). A higher prevalence of *Lactobacillus* has the potential to support nutrient absorption and energy availability, both of which are crucial for elite athletes (47). In contrast, the greater abundance of *Bacteroides* and *Prevotella* in HAsub indicates a microbiota adapted to a fiber-rich diet supporting carbohydrate metabolism and gut health (48). These distinct microbial profiles likely mirror differing physiological and dietary demands of athletes and healthy adults, with athletes adapted to heightened physical and metabolic stress.

### Linear discriminant analysis effect size analysis identified differences in microbial taxa

LEfSe analysis indicated enrichment of bacteria from the *Escherichia-Shigella* group in ATHsub. Due to their high genetic similarity (>90% sequence homology) to enteropathogenic *Escherichia coli*, *Shigella* species cannot be reliably distinguished on the basis of 16S rRNA sequencing, often resulting in their classification as a single cluster. An increased abundance of *E. coli* in athletes may reflect a potential training- and lifestyle-related response to high metabolic demands, oxidative stress regulation, and dietary factors, particularly protein intake (49). Nevertheless, some strains of the *Escherichia-Shigella* group exhibit opportunistic pathogenicity and pro-inflammatory effects (50, 51). A higher abundance of *Escherichia-Shigella* in athletes may also indicate increased demands for vitamin K synthesis, which plays a crucial role in regulating blood coagulation, bone and muscle health (52), as well as inflammation control (53). These findings may reflect a compensatory microbial adaptation to frequent physical stress, a hypothesis that warrants further investigation. *Faecalibacterium sp. UBA1819*, another taxon enriched in ATHsub, remains relatively understudied. It is well established that the genus *Faecalibacterium* is a major producer of SCFAs, which support intestinal health and immune balance (54). However, further studies are needed to determine whether these bacteria exhibit specialized metabolic functions, which might be relevant for athletic training and specific dietary needs. In contrast, the HAsub group exhibited significant enrichment in *Roseburia, Lachnospira,* and *Parasutterella*, genera commonly associated with a fiber-rich diet, gut health, and metabolic stability (55–57). However, the taxa enriched in ATHsub highlight the potential trade-offs of elite athletic performance, where microbiota may prioritize rapid energy utilization, metabolic efficiency, and recovery, possibly at the expense of microbial stability. These findings align with the hypothesis that the gut microbiota of elite athletes is specialized to meet the metabolic and physiological demands of their unique lifestyle, while healthy non-athletic individuals exhibit a more conventional microbial profile suited for long-term stability and health (47).

### Predictive functional profiling analysis

All functional pathway analyses are based on predictive metagenomic inference and should therefore be interpreted as hypothesis-generating rather than confirmatory. The increased prevalence of amino acid biosynthesis pathways in ATHsub, particularly for L-tryptophan, L-arginine, and the superpathway of histidine, purine, and pyrimidine, suggests a microbial adaptation favoring enhanced protein synthesis and nitrogen balance regulation. Tryptophan metabolism plays a crucial role in neurotransmitter synthesis, including serotonin, and immune modulation, such as the kynurenine pathway, both of which are critical for performance and recovery (58). Similarly, histidine serves as a precursor for histamine, which influences vasodilation and inflammatory responses, while arginine degradation is involved in nitric oxide (NO) production, a key regulator of vascular function and muscle efficiency (59). These microbial signatures associated with energy metabolism may support increased muscle protein turnover and enhanced endurance capacity in elite athletes. It is also evident that HAsub have higher functional capacities for essential amino acids such as lysine, isoleucine, valine, and histidine. This may indicate that ATHsub have higher amino acid demands, resulting in a reduced availability of these amino acids as an educt, therefore limiting these functional capacities (60).

The increased functional prevalence of glycolysis pathways in ATHsub suggests a microbiome adapted to enhanced glucose utilization and storage, potentially supporting sustained exercise performance. Glycogen serves as a crucial energy reserve during prolonged muscular activity, and efficient microbial glycogen metabolism may enhance host energy homeostasis (61). The enrichment of the fatty acid β-oxidation pathways underlines this, since it also supports long-lasting muscular activity. Both findings could indicate improved substrate flexibility, which is a crucial factor for athletic performance (62).

The enrichment of pathways related to microbial cell wall biosynthesis, including peptidoglycan and O-antigen building block biosynthesis, suggests potential microbiome adaptations to secure gut barrier integrity and immune balance. Both factors present a significant challenge for athletes, as recurrent ischemia-reperfusion processes and the associated formation of reactive oxygen species place stress on the intestinal epithelium and activate pro-inflammatory pathways (63). The enrichment of predicted vitamin K2 (menaquinone) and ubiquinone biosynthesis pathways in athletes is intriguing and may reflect a potential training- and lifestyle-related adaptive host-microbiome interaction. However, these findings must be interpreted with caution, given the predictive nature of the analysis. Prior work has suggested roles of microbiome-derived vitamin K in vascular and metabolic health, and Coenzyme Q has been studied in the context of mitochondrial function and exercise performance (64). Nevertheless, age differences and uncontrolled supplementation practices may have influenced these results. Therefore, we present these functional predictions as hypothesis-generating observations that warrant validation by targeted metabolomic or intervention studies. In this context, it is noteworthy that mitochondria share an evolutionary origin with α-proteobacteria (65), making it plausible that microbial metabolites, including menaquinones and ubiquinones, influence mitochondrial efficiency and oxidative metabolism in host tissues. This aligns with evidence suggesting that gut microbial communities in athletes may shift toward metabolic pathways linked to energy production and immune regulation (5). Moreover, menaquinones, as vitamin K2 derivatives, serve as essential cofactors in bacterial respiration (66). Our data indicate an increased predicted abundance of menaquinone-producing bacteria in ATHsub, particularly in biosynthetic pathways for menaquinol-7, -8, and -9. While this may represent a training- and lifestyle-related microbial response to the higher metabolic turnover and oxidative demands in athletes (49, 67), such interpretations remain speculative until validated by direct metabolomic measurements or intervention studies. It is important to emphasize that all functional pathway results are based on predictive metagenomic inference from 16S rRNA gene profiles rather than direct metagenomic or metabolomic measurements. PICRUSt2 predictions depend on reference genome availability and do not provide quantitative information on metabolite concentrations. Consequently, the functional differences reported here should be regarded as hypothesis-generating and require validation using shotgun metagenomics or targeted metabolomic approaches.

Compared with previous studies in athletic populations, the present work provides several distinct contributions. First, by integrating junior and senior elite athletes, we were able to disentangle age-related diversity patterns from training-associated microbial features. Second, the matched-subgroup design controlling for sex, age, BMI, and diet reduces confounding effects that have limited comparability in earlier cohort studies. Third, the combined taxonomic and predicted functional analyses consistently point toward microbial adaptations related to energy metabolism, oxidative stress regulation, and quinone biosynthesis, extending prior observations that focused primarily on compositional changes. Together, these aspects position the present study as a complementary and hypothesis-generating extension of existing athlete microbiome research rather than a replication of earlier findings.

### Limitations and future directions

This study has several limitations. Although standardized laboratory protocols and bioinformatic pipelines were applied across all samples, sequencing was performed in separate batches and across different runs. In the matched subgroup, ATHsub samples were distributed across multiple sequencing runs, whereas HAsub samples originated from a separate batch; thus, sequencing run/batch was structurally confounded with cohort/project of origin. Although processing showed partial temporal interspersion, technical batch/run effects cannot be disentangled from biological group differences in this subgroup and therefore cannot be excluded. Accordingly, ATH-versus-HA subgroup comparisons should be interpreted with appropriate caution. In addition, the inclusion of athletes from multiple Olympic disciplines reflects real-world elite sport but introduces heterogeneity in training modalities and physiological demands, which may increase inter-individual variability and attenuate discipline-specific microbiome signals. Sport-specific subgroup analyses were therefore not performed due to limited statistical power. The absence of detailed training load and cardiorespiratory fitness data, along with slight differences in dietary assessment tools across cohorts, may constrain the precision in attributing microbiome shifts to training-specific factors. Age variability and differences in body composition may also have introduced residual confounding. Functional inferences were based on PICRUSt2 predictions, which depend on reference genome databases and may not fully capture the metabolic complexity of the gut microbiota (26). Moreover, other lifestyle-related factors were not systematically assessed, although these are known modulators of the gut microbiome (15). Supplement use, particularly micronutrient supplementation, may represent a residual confounder that could have influenced functional predictions and should be addressed in future studies. Notably, it was demonstrated that microbial composition in athletes can be altered by differences in dietary quality alone, even when comparable intakes of macronutrients and fiber are observed (68). Thus, unmeasured dietary and lifestyle factors may have contributed to the observed microbial signatures. Finally, the cross-sectional design precludes causal inference; longitudinal studies integrating metabolomics and metatranscriptomics will be necessary to validate functional implications and to capture the dynamic interplay between training phases, diet, and the microbiome.

### Conclusion

In conclusion, we identified distinct microbial signatures in elite athletes compared with matched controls, including enrichment of genera such as *Escherichia-Shigella* and *Faecalibacterium* sp. UBA1819, and predicted functional shifts toward menaquinone and ubiquinone biosynthesis pathways. These associations may indicate potential host-microbiome interactions relevant to energy metabolism and oxidative stress regulation but must be interpreted with caution given the predictive nature of functional analyses. Moreover, methodological limitations, including the broad age range of participants, differences in dietary assessment methods, and the lack of detailed training load and physiological status data, should be acknowledged. Collectively, our findings provide a foundation for hypothesis-generating investigations into the potential role of the gut microbiome in supporting elite athletic performance, highlighting the need for carefully controlled, longitudinal studies integrating dietary monitoring, training load assessment, and direct metabolomic or functional assays. A comprehensive understanding of the bidirectional interactions occurring between gut microbiota and host metabolism is crucial for the development of effective microbiome-targeted interventions within the domain of sports science. Future research integrating microbiome sequencing and metabolomics will be essential to elucidate the precise role of the gut microbiome in optimizing human performance, particularly through longitudinal and intervention studies that incorporate detailed training load assessment and link microbial functional signatures to physiological and recovery-related outcomes.

## Supplementary Material

Reviewer comments

## Data Availability

The raw 16S rRNA gene sequencing data have been deposited in the NCBI Sequence Read Archive (SRA) under BioProject accession number PRJNA1259926. The associated metadata file can be found at https://doi.org/10.5281/zenodo.20640491.

## SUPPLEMENTAL MATERIAL

OPEN PEER REVIEW reviewer-comments.pdf An accounting of the reviewer comments and feedback.

ASM does not own the copyrights to Supplemental Material that may be linked to, or accessed through, an article. The authors have granted ASM a non-exclusive, world-wide license to publish the Supplemental Material files. Please contact the corresponding author directly for reuse.
