# Supplementary material for: Signatures in the gut microbiome of German elite athletes: insights from a matched-subgroup analysis
Source: mSystems. 2026 Jun 30;11(7):e00489-26. doi: 10.1128/msystems.00489-26 (PMC13387018; doi:10.1128/msystems.00489-26)
Supplement: Reviewer comments [file reviewer-comments.pdf]

AMERICAN SOCIETY FOR MICROBIOLOGY | mSystems®

Signatures in the Gut Microbiome of German Elite Athletes: Insights from a Matched-Subgroup Analysis

Claudia Lenz, Waldemar Seel, Theresa Dombrowski, Sebastian Hacker, Marie-Christine Simon, Karen Zentgraf, Christine Dawczynski, and Karsten Krueger

Corresponding Author(s): Karsten Krueger, Justus-Liebig-Universitat Giessen

Review Timeline:

Submission Date:	April 17, 2026
Editorial Decision:	May 20, 2026
Revision Received:	June 1, 2026
Accepted:	June 3, 2026

Editor: Fatima Pereira

Reviewer(s): Disclosure of reviewer identity is with reference to reviewer comments included in decision letter(s). The following individuals involved in review of your submission have agreed to reveal their identity: Bhumika Dubay (Reviewer #2)

Transaction Report:

(Note: With the exception of the correction of typographical or spelling errors that could be a source of ambiguity, letters and reports are not edited. The original formatting of letters and referee reports may not be reflected in this compilation.)

DOI: https://doi.org/10.1128/msystems.00489-26

Re: mSystems00489-26 (Signatures in the Gut Microbiome of German Elite Athletes: Insights from a Matched-Subgroup Analysis)

Dear Prof. Karsten Krueger:

Thank you for the privilege of reviewing your work. Below you will find my comments, instructions from the mSystems editorial office, and the reviewer comments.

Authors are encouraged to comply with community metadata standards, such as the "Minimal Information about any (X) Sequence" (MIxS) checklist, when submitting to GenBank, SRA, ENA, or DDBJ. Please upload a table with core and type-specific descriptors for all samples: https://www.gensc.org/pages/standards/checklists.html

Please return the manuscript within 60 days; if you cannot complete the modification within this time period, please contact me. If you do not wish to modify the manuscript and prefer to submit it to another journal, notify me immediately so that the manuscript may be formally withdrawn from consideration by mSystems.

Revision Guidelines
To submit your modified manuscript, log into the submission site at https://msystems.msubmit.net/cgi-bin/main.plex. Go to Author Tasks and click the appropriate manuscript title to begin. The information you entered when you first submitted the paper will be displayed; update this as necessary. Note the following requirements:

• Upload point-by-point responses to the issues raised by the reviewers in a file named "Response to Reviewers," NOT in your cover letter.
• Upload a compare copy of the manuscript (without figures) as a "Marked-Up Manuscript" file.
• Upload a clean .DOC/.DOCX version of the revised manuscript and remove the previous version.
• Each figure must be uploaded as a separate, editable, high-resolution file (TIFF or EPS preferred), and any multipanel figures must be assembled into one file.
• Any supplemental material intended for posting by ASM should be uploaded with their legends separate from the main manuscript. You can combine all supplemental material into one file (preferred) or split it into a maximum of 10 files with all associated legends included.

For complete guidelines on revision requirements, see our Submission and Review Process webpage. Submission of a paper that does not conform to guidelines may delay acceptance of your manuscript.

Data availability: ASM policy requires that data be available to the public upon online posting of the article, so please verify all links to sequence records, if present, and make sure that each number retrieves the full record of the data. If a new accession number is not linked or a link is broken, provide mSystems production staff with the correct URL for the record. If the accession numbers for new data are not publicly accessible before the expected online posting of the article, publication may be delayed; please contact production staff (mSystems@asmusa.org) immediately with the expected release date.

Publication Fees: For information on publication fees and which article types are subject to charges, visit our website. If your manuscript is accepted for publication and any fees apply, you will be contacted separately about payment during the production process; please follow the instructions in that e-mail. Arrangements for payment must be made before your article is published.

ASM Membership: Corresponding authors may join or renew ASM membership to obtain discounts on publication fees. Need to upgrade your membership level? Please contact Customer Service at Service@asmusa.org.

The ASM Journals program strives for constant improvement in our submission and publication process. Please tell us how we can improve your experience by taking this quick Author Survey.

Thank you for submitting your paper to mSystems.

Sincerely,
Fatima Pereira
Editor
mSystems

Reviewer #2 (Comments for the Author):

The authors have addressed my concerns satisfactorily and improved the manuscript substantially. The revised terminology

around the matched-subgroup analysis is clearer, and the limitations related to sequencing batch effects and exploratory LEfSe findings are now more appropriately acknowledged. I have no further substantive concerns. Please perform a final spell check and formatting check, including special characters such as α, β, and γ, before publication.

Response to Reviewers

Manuscript: *Signatures in the Gut Microbiome of German Elite Athletes*

We sincerely thank **Reviewer 2** for the constructive and highly insightful comments. We greatly appreciate the positive assessment of the revised manuscript and the comments regarding formatting and spell check.

All changes are described in detail below:

"The authors have addressed my concerns satisfactorily and improved the manuscript substantially. The revised terminology around the matched-subgroup analysis is clearer, and the limitations related to sequencing batch effects and exploratory LEfSe findings are now more appropriately acknowledged. I have no further substantive concerns.

Please perform a final spell check and formatting check, including special characters such as α, β, and γ, before publication."

Our Response:

We carefully reviewed the manuscript for spelling and formatting errors. With respect to language, the manuscript was systematically revised to ensure consistent use of American English throughout, including correction of spelling variants (e.g., "fecal", "fiber"). Tense inconsistencies in the Results and Discussion sections were resolved, with past tense applied consistently when describing the study's own findings. Serial commas were applied throughout in accordance with journal style.

As mentioned from Reviewer 2, alpha- and beta-diversity are now consistently described, and special characters (such as α, β, and γ) have been checked throughout the manuscript, tables and figures. Figure 6C has therefore been amended and the minor formatting error corrected; it is now titled 'Fatty acid beta-oxidation I'.

In the Tables and Figures: the group label in Table 2 was corrected from "ATHsub/HAsub" to "ATH/HA," consistent with the full-cohort PERMANOVA comparison (n = 251) described in both the table caption and the Results text. A missing semicolon in the post-hoc column of Table 1 was added.

Finally, the references were comprehensively reviewed, and minor additions (e.g., page numbers or volume) were added.

The raw 16S rRNA gene sequencing data have been deposited in the NCBI Sequence Read Archive (SRA) under BioProject accession number: PRJNA1259926 (Link: http://www.ncbi.nlm.nih.gov/bioproject/1259926) The manuscript includes all the necessary information.

We thank Reviewer 2 once more for the constructive feedback. We believe that the revised version now fully aligns with the expectations for publication with mSystems.

Re: mSystems00489-26R1 (Signatures in the Gut Microbiome of German Elite Athletes: Insights from a Matched-Subgroup Analysis)

Dear Prof. Karsten Krueger:

Your manuscript has been accepted, and I am forwarding it to the ASM production staff for publication. Your paper will first be checked to make sure all elements meet the technical requirements. ASM staff will contact you if anything needs to be revised before copyediting and production can begin. Otherwise, you will be notified when your proofs are ready to be viewed.

Data Availability: ASM policy requires that data be available to the public upon online posting of the article, so please verify all links to sequence records, if present, and make sure that each number retrieves the full record of the data. If a new accession number is not linked or a link is broken, provide production staff with the correct URL for the record. If the accession numbers for new data are not publicly accessible before the expected online posting of the article, publication may be delayed; please contact ASM production staff immediately with the expected release date.

Publication Fees: For information on publication fees and which article types have charges, please visit our website. We have partnered with Copyright Clearance Center (CCC) to collect author charges. If fees apply to your paper, you will receive a message from no-reply@copyright.com with further instructions. For questions related to paying charges through RightsLink, please contact CCC at ASM_Support@copyright.com or toll free at +1-877-622-5543. CCC makes every attempt to respond to all emails within 24 hours.

ASM Membership: Corresponding authors may join or renew ASM membership to obtain discounts on publication fees. Need to upgrade your membership level? Please contact Customer Service at Service@asmusa.org.

Embargo Policy: A press release may be issued as soon as the manuscript is posted on the mSystems Latest Articles webpage. The corresponding author will receive an email with the subject line "ASM Journals Author Services Notification" when the article is available online.

Cover Image Submissions: If you would like to submit a potential Cover Image, please email a file and a short legend to msystems@asmusa.org. Please note that we can only consider images that (i) the authors created or own and (ii) have not been previously published. By submitting, you agree that the image can be used under the same terms as the published article. Image File requirements: TIF/EPS, 7.5 inches wide by 8.25 inches tall (at least 2,250 pixels wide by 2,475 pixels tall), minimum 300 dpi resolution (600 dpi preferred), RGB, and no figure elements, e.g., arrows or panel labels. The legend should be a short description of the image, 1-2 sentences recommended. Please download and use this interactive template in Adobe to ensure that your proposed cover image meets our size requirements (https://journals.asm.org/pb-assets/pdf-text-excel-files/ASM-Interactive-Sizing-Cover-Template-1715689791.pdf).

Thank you for submitting your paper to mSystems.

Sincerely,
Fatima Pereira
Editor
mSystems
